# High Body Mass Index Reduces Glomerular Filtration Rate Decline in Type II Diabetes Mellitus Patients With Stage 3 or 4 Chronic Kidney Disease

**DOI:** 10.1097/MD.0000000000000041

**Published:** 2014-07-25

**Authors:** Wen-Hung Huang, Chao-Yu Chen, Ja-Liang Lin, Dan-Tzu Lin-Tan, Ching-Wei Hsu, Tzung-Hai Yen

**Affiliations:** Department of Nephrology and Division of Clinical Toxicology, Chang Gung Memorial Hospital, Linkou Medical Center (WHH, CYC, JLL, DTLT, CWH, THY); and Chang Gung University and School of Medicine (WHH, CYC, JLL, DTLT, CWH, THY), Taoyuan, Taiwan, Republic of China (ROC).

## Abstract

Whether high body mass index (BMI) has an effect on progressive diabetic nephropathy in type II diabetes mellitus (DM) patients with chronic kidney disease (CKD) stage 3 or 4 remains unclear. This prospective study aimed to investigate the relationship between BMI and progression of renal function deterioration in type II DM patients with CKD stage 3 or 4.

A total of 105 type II DM patients with CKD stage 3 or 4 participated in this 24-month prospective observational study. Patients were divided into 3 groups according to BMI as follows: normal group, BMI of 18.5–22.9 kg/m^2^; overweight group, BMI of 23–24.9 kg/m^2^; and obese group, BMI of ≥25 kg/m^2^. The primary end point was a 2-fold elevation in serum creatinine levels (measured twice with a 1-month interval) from baseline values, need for long-term dialysis, or death during the 24-month observation period.

In the linear regression analysis with the stepwise method, each 1 kg/m^2^ increase in BMI led to an increase of 0.32 mL min^−1^ × 1.73 m^−2^ in the estimated glomerular filtration rate (95% confidence interval, CI, 0.01–0.62; *P* = 0.04) during the 24-month study period. Moreover, multivariate Cox regression analysis showed that compared with the obese group, the normal BMI group (hazard ratio = 2.76, 95% CI : 1.27–6; *P* = 0.01) achieved the primary outcome after adjusting for other factors.

In this 24-month prospective observational study, we showed that BMI of ≥25 kg/m^2^ was a protective factor for renal function deterioration in type II DM patients with CKD stage 3 or 4.

## INTRODUCTION

Diabetes mellitus (DM) is recognized as a major risk factor for the development of chronic kidney disease (CKD). The natural course of diabetic nephropathy is generally characterized by a variable period of hyperfiltration followed by a progressive decline in the glomerular filtration rate (GFR), once overt proteinuria appears.^1^ The level of glycemic control, prevalence of hypertension and hyperlipidemia, and smoking habits are some of the environmental factors that have been shown to influence the risk of renal failure. Obesity-related glomerular disease is associated with high renal plasma flow (RPF), suggesting a state of renal vasodilatation involving the afferent arteriole^2–5^ and defined morphologically as glomerulomegaly with/without focal segmental glomerulosclerosis.^6^ In several studies,^2,5,7^ obesity and DM have been found to exert a synergic effect on the rate of renal function decline. Nonlinear synergistic relationships may exist among many obesity-related disorders such as DM, hyperlipidemia, and hypertension, resulting in increased risk of cardiovascular disease (CVD). Moreover, several studies^8–11^ have shown that in patients undergoing hemodialysis, high body mass index (BMI) was a negative predictor of mortality. However, this observation is still controversial. To the best of our knowledge, studies on chronic renal disease such as CKD stage 3 or 4, and the effect of BMI on renal function are limited, particularly with regard to DM patients. Therefore, the aim of this study was to examine the effect of BMI on renal function by assessing the GFR of type II DM patients with CKD stage 3 or 4 in a 24-month study period.

## STUDY POPULATION AND METHODS

### Participants

This study complied with the guidelines of the Declaration of Helsinki and was approved by the medical ethics committee of Chang Gung Memorial Hospital, a tertiary referral center located in the northern part of Taiwan. Written informed consent was obtained from every participant, and the study was approved by the institutional review board of the Chang Gung Memorial Hospital. In addition, all individual information was securely protected (by delinking identifying information from main data set) and was only available to investigators. Furthermore, all the data were analyzed anonymously. All medical records during the study period, including medical history, laboratory data, and inclusion and exclusion factors were reviewed by 3 nephrology specialists (WHH, CWH, and THY). Finally, all the primary data were collected according to the Strengthening the Reporting of Observational Studies in Epidemiology guidelines.

This is a random-selection, prospective observational study on type II DM patients with CKD stage 3 or 4 in a single center. Recruitment started in January 2009, and follow-up ended in December 2011. Patients aged 30–83 years with type II DM with nephropathy and who received follow-up care at Chang Gung Memorial Hospital for >1 year were eligible for this study; eligible patients were requested to provide informed consent to participate in this study if they met the following criteria^12^: abnormal serum creatinine (SCr) level (>1.4 mg/dL), stage 3 or 4 CKD (estimated GFR, eGFR, between 15 mL min^−1^ × 1.73 m^−2^ and 60 mL min^−1^ × 1.73 m^−2^), diabetic retinopathy treated with or without laser therapy, daily urinary protein excretion of >0.5 g/d, absence of microhematuria on urine testing, normal-sized kidneys determined by echogram, and >5-year history of diabetes. Diabetic nephropathy was diagnosed based on renal histological examination findings in cases where renal biopsies were performed. The exclusion criteria were as follows: type I DM; renal insufficiency with a potentially reversible cause such as malignant hypertension and urinary tract infection; BMI < 18.5 kg/m^2^; hypercalcemia or drug-induced nephrotoxicity; presence of other systemic diseases such as connective tissue diseases; use of drugs that might alter the course of renal disease such as nonsteroidal anti-inflammatory agents, steroids, immunosuppressive drugs, or Chinese herbal drugs; drug allergies; and the absence of informed consent. The blood pressure of each patient was maintained at <140/90 mmHg with diuretics and angiotensin-converting-enzyme inhibitors (ACEIs) or angiotensin II receptor antagonists (ARAs), with or without calcium-blocking agents and/or vasodilators. Calcium carbonate was used to maintain the phosphate levels. None of the patients received vitamin D_3_ supplements because they had parathyroid hormone levels of <200 pg/mL. Each patient received dietary consultation. A diabetic diet (35 Kcal kg^−1^ d^−1^) with normal protein intake (0.8–1.0 g high-biological-value protein kg^−1^ d^−1^) was recommended to each patient. A nutritionist reviewed the dietary intake of each patient every 3–6 months. The patients also underwent a 24-hour urea excretion test every 3 months to ensure the nitrogen balance and dietary compliance.^13^

### Study Protocol

SCr level, glycosylated hemoglobin (HbA1c) level, daily urine protein excretion, daily protein intake (DPI), mean arterial pressure (MAP), cholesterol level (including high-density lipoprotein, HDL and low-density lipoprotein, LDL), and triglyceride (TG) level were measured using an autoanalyzer system (model 736; Hitachi, Tokyo, Japan) at the beginning and end of the study and every 6 months during the 24-month clinical observation period. Blood pressure and BMI were also measured at 6-month intervals. Renal function was assessed in terms of eGFR (mL min^−1^ 1.73 m^−2^ of body surface area). A modified eGFR equation for Chinese patients with type II diabetes (D-GFR) was used^14^ (mL/min/1.73 m^2^) (R^2^ = 0.95) : 313 × (Age)^−0.494^ (years) × [SCr]^−1.059^ (mg/dL) × [albumin]^+0.485^ (g/dL) for men, and 783 × (Age)^−0.489^ (years) × [SCr]^−0.877^ (mg/dL) × [serum urea nitrogen]^−0.150^ (mg/dL) for women. The BMI was calculated as [mass (kg)]/[height (m)]^2^. A total of 105 patients met the inclusion criteria and were classified into the following 3 groups according to their BMI, as defined by the Western Pacific Regional Office (WPRO) criteria^15,16^: normal BMI group, 30 normal-weight patients with BMI of 18.5–22.9 kg/m^2^; overweight group, 22 overweight patients with BMI of 23–24.9 kg/m^2^; and obese group, 53 obese patients with BMI of ≥25 kg/m^2^.

### Outcome Measures

The primary end point was a 2-fold elevation in SCr level (measured twice, 1 month apart) from the baseline values, need for long-term dialysis, or death during the 24-month observation period.

### Statistical Analysis

The Kolmogorov–Smirnov test showed all variables to be normally distributed. A *P* value > 0.05 was required to assume a normal distribution. All the tested variables in the different groups were assumed as normal distribution. The data were presented as mean ± SD for variables with normal distribution. χ^2^ test and one-way analysis of variance was performed to compare the clinical variables among the 3 groups. Generalized estimating equation (GEE) with linear analysis was used for longitudinal multivariate analysis to further assess the changes in variables over time and their association with renal function (eGFR) during the observation period. Moreover, multivariate Cox analysis was used to determine the significance of the baseline variables in predicting the primary end point during the study period. These models included all variables identified in the literature as related to the progression of diabetic nephropathy.^12,14,17–19^ All the nominal variables in linear regression were dummy coding transformed. Missing data was approached with listwise deletion. A *P* value < 0.05 was set as statistically significant. Data were analyzed using the Statistical Analysis System statistical software (version 6.12; SAS Institute, Cary, NC).

## RESULTS

### Study Subjects

A total of 105 (68 men and 37 women) patients participated in the 24-month-long study. Table 1 summarizes the demographic data, baseline chronic disease conditions, use of ACEIs or ARAs, and daily urinary protein levels for participants in each group. In the obesity group, the patients had the lowest HDL level (39.13 ± 7.48 mg/dL, *P* = 0.04), highest TG level (239.03 ± 194.5 mg/dL, *P* = 0.04), highest MAP (99.91 ± 10.0 mmHg, *P* = 0.01), and lowest DPI (0.93 ± 0.15, *P* = 0.04). However, age and levels of SCr, TG, LDL, HbA1c, and daily urine total protein were not significantly different among the 3 groups. In addition, 70 patients (66.6%) had hyperlipidemia and 101 patients (96.1%) had hypertension, and they were administered ACEIs or ARAs; 16 patients (15.2%) smoked. Among all the study patients, 34 (32.3%) had a history of CVDs, namely myocardial infarction, congestive heart failure, stroke, and diabetic foot. The obese group had the highest number of patients with hyperlipidemia (*P* < 0.01) and CVD (*P* = 0.02).

**TABLE 1:**
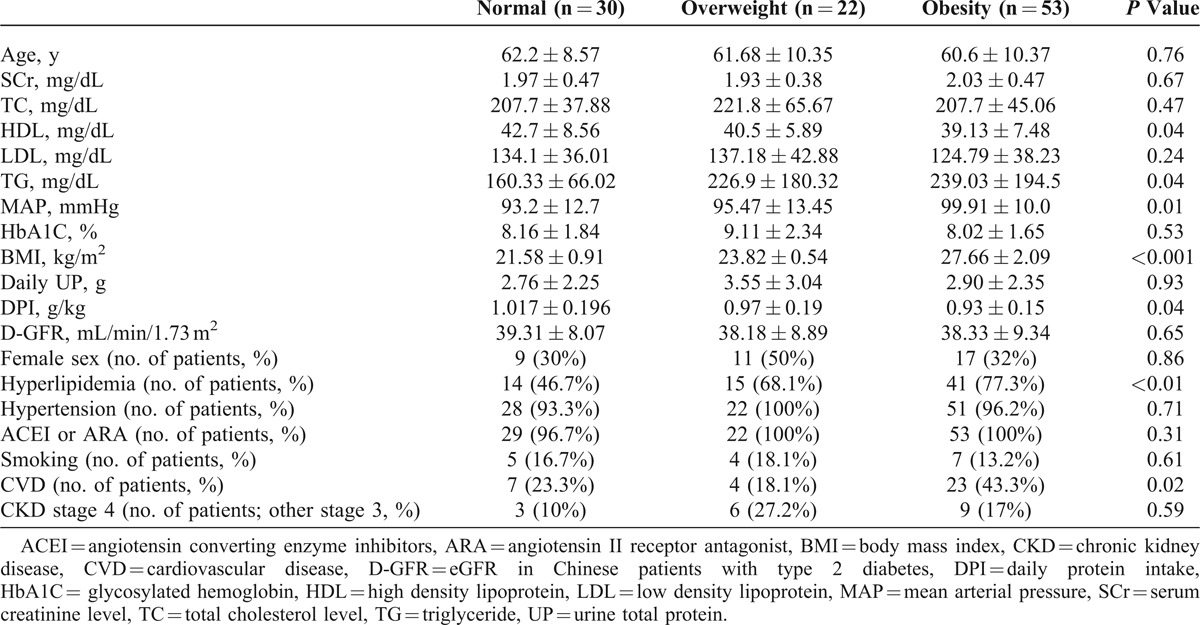
Baseline Characteristics and Comparison of the 105 Patients

### Longitudinal Follow-Up for 24 Months

**FIGURE 1:**
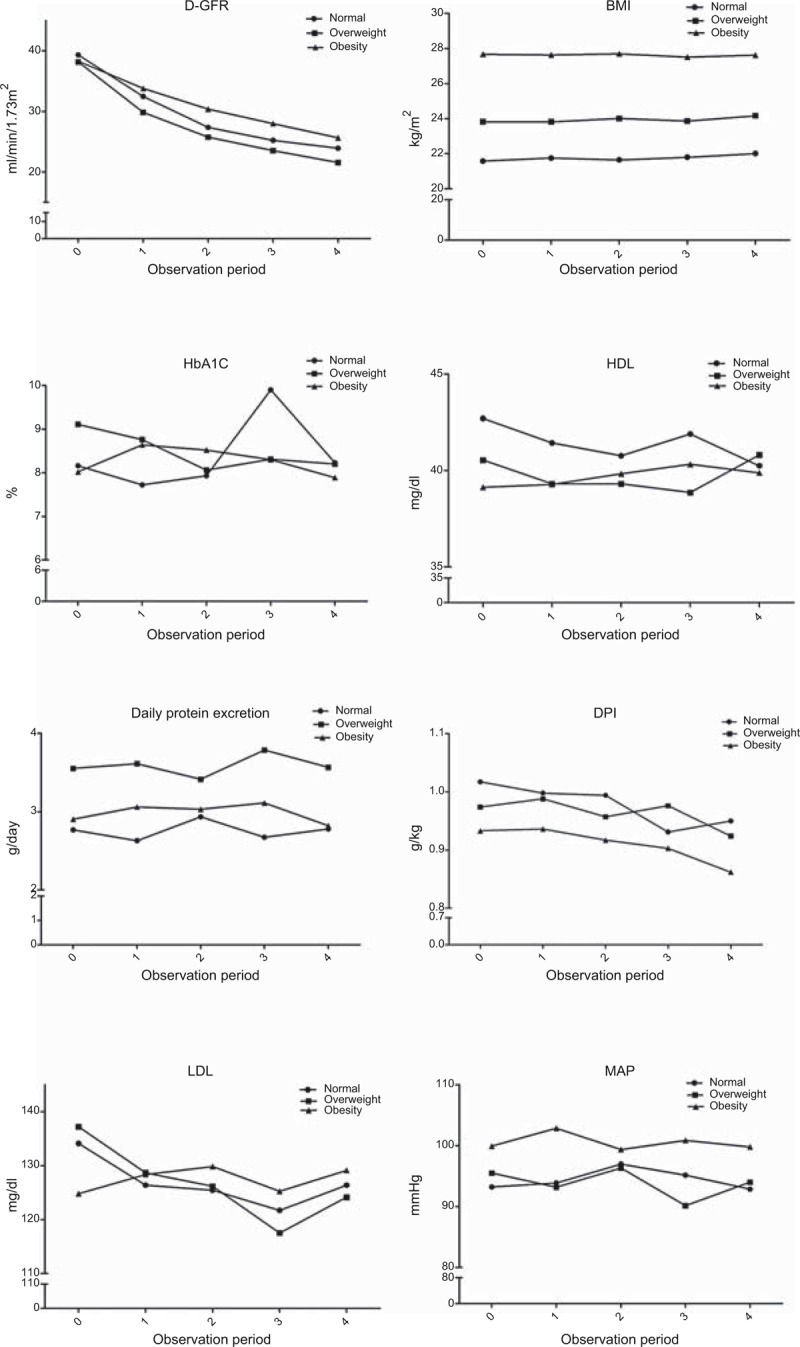
The trend of changes in D-GFR, MAP, DPI, and levels of HDL, HbA1c, and daily proteinuria in 24 months. During the 24-month study period, the D-GFR showed a trend of decline among the 3 groups. The rate of GFR decline was the least in the obese group. Every unit on the x-axis represents a duration of 6 months. BMI = body mass index, D-GFR = eGFR in Chinese patients with type 2 diabetes, DPI = daily protein intake, HbA1c = glycosylated hemoglobin, HDL = high density lipoprotein, LDL = low density lipoprotein, MAP = mean arterial pressure.

The trend of changes in the D-GFR, BMI, MAP, DPI, and levels of LDL, HDL, HbA1c, and daily proteinuria during the 24-month study period are shown in Figure 1. Of note, in the obese group, decrease in the rate of D-GFR was the lowest among the 3 groups; the 24-hour urine total protein level did not show any significant changes between the groups.

### Outcome Measures

In this study, the number of patients reaching the outcome measures in normal, overweight, and obesity groups were 13, 12, and 19, respectively. For examining the influence of clinical features on the changes in D-GFR in 24 months, we used linear regression analysis to evaluate the association between changes in D-GFR and the clinical variables. GEE with linear analysis showed that BMI predicted the progression of eGFR, after adjusting for other variables (Table 2). Each 1 kg/m^2^ increase in BMI led to an increase of 0.32 mL min^−1^ × 1.73 m^−2^ in eGFR (95% confidence interval, CI, 0.01–0.62; *P* = 0.04) during the 24-month study period. Moreover, multivariate Cox regression analysis showed that compared with the obese group, the normal BMI group (hazard ratio = 2.76, 95% CI, 1.27–6; *P* = 0.01) achieved the primary outcome, after adjusting for other factors (Table 3). Additionally, with an increase in BMI, the primary outcome showed a decrease (*P* for trend = 0.03).

**TABLE 2:**
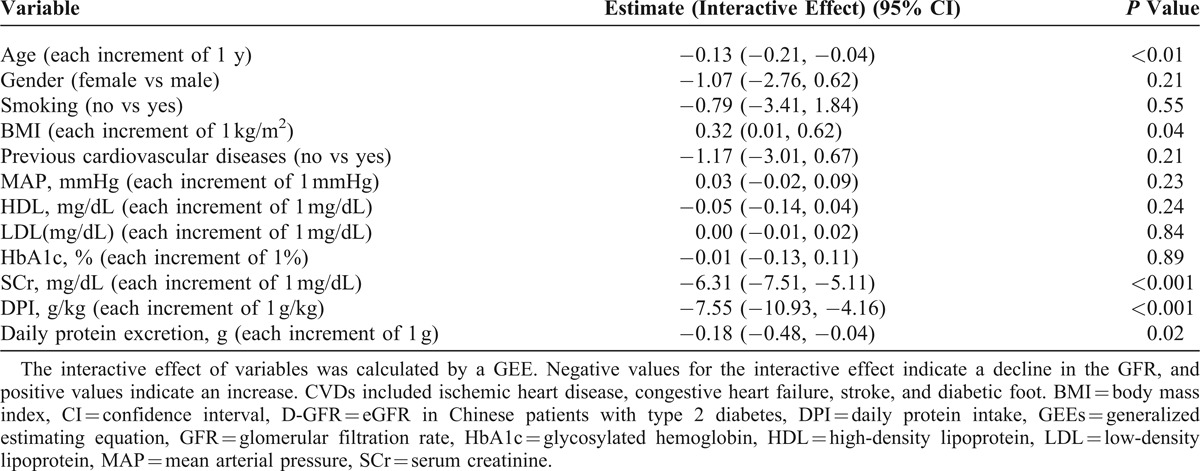
Longitudinal Multivariate Analysis of Clinical Predictors of Progressive Change in the eGFR (D-GFR) Using GEEs During the 24-Month Longitudinal Study Period (N = 105)

**TABLE 3:**
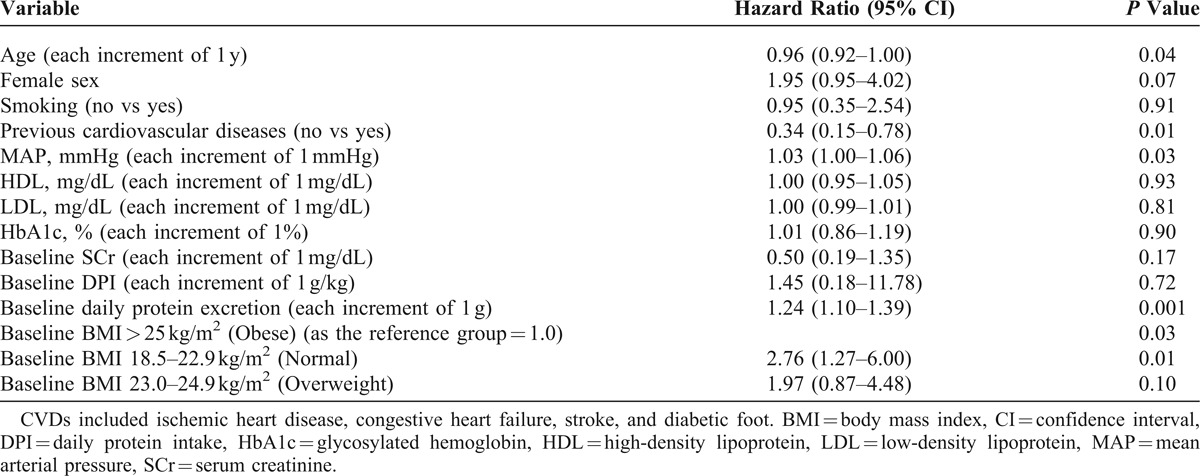
Cox Regression Analysis of the Overall Risk of the Primary Outcome of Progressive Renal Insufficiency According to Baseline Prognostic Factors (N = 105)

## DISCUSSION

In this 24-month prospective observational study, we found that in type II DM patients with CKD stage 3 or 4, a BMI of >25 kg/m^2^ was a protective factor for renal function deterioration.

Globally, diabetes is recognized as a major risk factor for the development of CKD. The natural history of DM nephropathy is generally characterized by a variable period of hyperfiltration followed by progressive GFR decline, once overt proteinuria appears.^1,20^ The initial glomerular hyperfiltration characteristic of diabetes leads to relatively low SCr concentrations in the patients, and the early decline in the GFR leads to undetectable changes in the SCr concentration.^21^ Nelson et al^1^ pointed out that there are 3 general classes of GFR decline in type II DM patients: linear decline, bimodal decline, and variable decline. The differences in the pattern of GFR decline due to diabetic nephropathy are probably influenced by a number of different genetic and environmental factors. The level of glucose control, degree of hypertension and hyperlipidemia, and smoking habits are some of the environmental factors that have been shown to increase the risk of renal failure. However, studies on the effect of BMI in renal function protection in type II DM patients with CKD stage 3 or 4 are limited. In this study, the decline in GFR was the least in the obese group during the 24-month study period. It is interesting that the initial clinical condition of the obese group was the worst (low HDL level, high TG level, high MAP, and prevalence of CAD). Figure 1 also shows that the DPI of the obesity group was the least among the 3 groups but the difference was not significant (*P* > 0.05). The BMI of each group did not significantly change during the 24-month period. However, eliminating the effect of the interaction of clinical variables, in our advanced analysis, after adjusting for relative clinical variables (Table 2), the protective effect of high BMI on GFR decline, morbidity, and mortality were evident.

Obesity-associated hyperfiltration is associated with high RPF, suggesting a state of renal vasodilatation involving the afferent arteriole.^2–5^ Although the mediators involved in these early glomerular structural changes are unknown, neurohumoral factors such as angiotensin II, sympathetic stimulation, and changes in intrarenal pressure caused by high blood pressure and dilation of the afferent arterioles may play important roles in inducing these changes.^22^ Chagnac et al^2^ pointed out that with the transmission of increased arteriolar pressure through a dilated glomerular afferent arteriole, the resulting increased transcapillary pressure gradient leads to increased GFR. The number of nephrons does not increase with increasing body fat; therefore, obesity probably induces an increase in the GFR of individual nephrons.

The role of obesity in a healthy population and in patients with chronic illnesses is worthy of discussion.^23^ In a healthy person, the absence of adipocytes leads to metabolic dysfunction that is associated with insulin resistance, hyperlipidemia, and fatty liver.^24^ Obese individuals have high lipoprotein concentrations that counteract the inflammatory effects of circulating endotoxins.^25^ Of note, reduction in total body fat is associated with a decline in humoral immunity.^26^ Overweight and obese individuals have a high amount of muscle mass. This increased amount of lean body mass might play an additional protective role in catabolism.^27^ In contrast, adipose tissue is recognized as a source of inflammatory cytokines such as tumor necrosis factor-α, interleukin-6, and C-reactive protein, and obesity has been suggested to be a low-grade inflammatory condition.^28,29^ The circulating level of leptin, a peptide produced predominantly by adipocytes and fat mass, is found to be consistent with the amount of fat mass.^30^ Nevertheless, the net effect of leptin on vascular function remains unclear. In several studies, leptin-induced vasodilatation has been found to be associated with endothelium and nitric oxide-dependent pathways through the effects of vasodilatation on the endothelium.^31^ On the basis of the above points, we could hypothesize that initially, both obesity and DM may have synergic effects on renal function that consequently affect hyperfiltration and high RPF as well as the rate of renal function decline. However, in the case of CKD stage 3 or 4, after majority of the nephrons undergo sclerosis, the complex mechanisms of obesity may maintain the surviving nephrons in a condition of glomerular hyperperfusion and hyperfiltration through a dilated glomerular afferent arteriole that consequently leads to the relatively high GFR that we observed in this study.

Of note, in this study, the obese patients were on a low-protein diet (LPD) during the observation period (Figure 1). The effect of dietary protein restriction on the progression of CKD remains controversial.^32–34^ However, when the outcome criterion used is renal death or time for renal replacement therapy, most studies showed a positive effect of protein restriction. In a meta-analysis, Fouque and Aparicio^35^ found that a reduction in protein intake by 0.2 g kg^−1^ d^−1^ was associated with reduced renal death by approximately 40%. In 2009, in another meta-analysis, the relative risk of renal death was 0.7 in favor of a reduction in protein intake.^36^ Brunori et al^37^ found that patients aged 70 years on supplemented very-low-protein diet (SVLPD) showed delayed initiation of dialysis by approximately 11 months without any negative consequence on morbidity and mortality. In comparison with LPD, SVLPD seems to have an additive effect; Chang et al^38^ studied 120 patients who were trained for 6 months and were on an LPD (0.6 g kg^−1^ d^−1^) and then switched to an SVLPD. The GFR decline during the SVLPD period was significantly lower in patients with and without diabetes. In our study, the obese group patients had the lowest DPI (0.8–0.9 g kg^−1^ d^−1^) throughout the 24-month study period; therefore, protein restriction in obese patients had a protective effect on GFR decline (Table 2).

Currently, to the best of our knowledge, there are no studies focusing on the effect of BMI on renal function in type II DM patients with CKD stage 3 or 4. Therefore, the relation between BMI and renal function in these patients is still unclear. Discussed on the end-stage renal disease patients requiring dialysis, Yen et al^39^ showed a survival advantage of high BMI in Taiwanese patients undergoing maintenance hemodialysis (MHD). Similarly, Johansen et al^11^ analyzed the US Renal Data System of >400,000 MHD patients and found that high BMI was associated with a survival advantage; the survival curve plateaued at a BMI of ≥25 kg/m^2^. In Kalantar-Zadeh’s^40^ review, dialysis patients with BMI of ≥25 kg/m^2^ had a lower relative risk of death than patients with BMI of <25 kg/m^2^. Kalantar-Zadeh^40^ stated that a possible explanation for the survival advantage of high BMI is the time discrepancy among competitive risk factors: overnutrition versus undernutrition. Underweight patients may experience malnutrition–inflammation complex syndrome (MICS);^41–43^ MICS refers to a state of undernutrition, which may predispose one to infection or other inflammatory conditions. Moreover, when individuals are malnourished and have a low BMI, they are highly susceptible to inflammatory diseases.^41,44^ Alternatively, obese patients may have an improved survival rate in the short-term but not necessarily in the long-term—Kaizu et al’s^45^ long-term study (>12 y) showed no obesity-survival superiority, whereas Yen’s short-term study (approximately 3 y) showed an obesity-survival superiority. Therefore, in the present 24-month-long study, from the Cox regression analysis (Table 3), after adjustment for relative risk factors, high BMI (BMI ≥25 kg/m^2^) was found to be a protective factor for the outcome measure. However, further long-duration studies are required to clarify whether high BMI has a favorable effect on the outcome measure in the studied population.

The cutoff points for obesity (BMI > 25.0 kg/m^2^) in this study followed the WPRO criteria, which should be explained. We know that the relationship between BMI and body fat deposit is different among ethnic groups.^15,16^ Asians have a higher body fat deposit at a lower BMI than white patients.^16^ The World Health Organization criteria for classifying obesity in white patients (BMI > 30.0 kg/m^2^) may not be suitable for Asians. For the above reason, WPRO has proposed the definition of obesity as BMI >25.0 kg/m^2^.

This study has some limitations. First, it was designed as a single-center study with small number of enrolled patients. However, these study-patients were randomly selected. Second, comparing the advantages and disadvantages of BMI with regard to mortality is difficult and complicated, particularly in type II DM patients with CKD stage 3 or 4 who are at a high risk of chronic comorbidities such as cerebral vascular disease, because many factors affect the pattern of renal function. Third, we did not measure the adiposity or the lean body mass of the patients directly such as the ratio of waist circumference to hip circumference or central adiposity. Fourth, this study was a 24-month-long observational study. The observation period may be too short for clarifying the disadvantage of obesity-induced metabolic disorders, because cardiovascular and cerebrovascular events did not occur in this study. Further long-duration follow-up studies are required for linking the aforementioned variables to mortality rates. Finally, we could not completely rule out the beneficial effects of CKD education on the progression of renal function. We did not know whether the patients had changed lifestyle or were indeed in compliance with educational content to protect kidney function.

## CONCLUSION

This prospective observational study showed that after adjustment for relative risk factors, obesity was a significant protective factor related to 2-year mortality and renal function deterioration in type II DM patients with CKD stage 3 or 4.

## ACKNOWLEDGMENTS

We thank the members of the Statistic Center in Chang Gung Memorial Hospital for their invaluable and dedicated assistance.
